# *In vitro* cell culture models for ultrasound treatments using collagen-based scaffolds

**DOI:** 10.1016/j.mex.2022.101909

**Published:** 2022-11-04

**Authors:** Sanjana Gopalakrishnan, Aarohi Gupta, Dorcas Matuwana, John J. Amante, Cathal J. Kearney, Vincent M. Rotello

**Affiliations:** a Department of Chemistry, University of Massachusetts Amherst, Amherst, MA 01003, United States; b Department of Biomedical Engineering, University of Massachusetts Amherst, Amherst, MA 01003, United States

**Keywords:** Ultrasound treatment, *in vitro* cell culture models, Collagen-based materials, 2D cell culture, 3D cell culture

## Abstract

Applications involving ultrasound treatment as a therapeutic strategy have gained interest due to its enhanced tissue penetration, broad availability, and minimal invasiveness. Recently, ultrasound treatment has been utilized for applications such as controlled drug delivery, enhanced drug penetration, sonodynamic therapy for generating ROS species, and targeted tissue ablation. However, our ability to study and explore applications is limited by the lack of *in vitro* models that enable efficient and representative screening of ultrasound-based therapeutic strategies. There is a need for cell culture approaches that mimic the mechanical environment of native tissues, which can prevent uncontrolled cell lysis due to ultrasonic energy. We developed two-dimensional and three-dimensional collagen-based materials for culturing cells *in vitro* that withstand ultrasound treatment. We hypothesized that the collagen matrix mimics the extracellular matrix and absorb most of the energy from ultrasound treatment – similar to *in vivo* effects – thereby preventing uncontrolled cell lysis. In this study, we developed a strategy for fabricating both the 2D coatings and 3D hydrogels coatings and tested the viability of the cultured cells post different durations of ultrasound treatment.

## Graphical abstract

**Figure:**
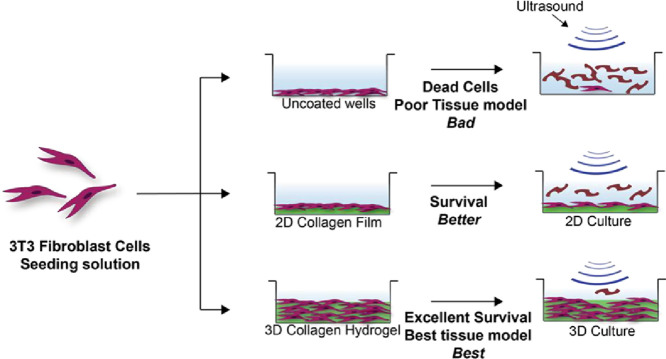

**Specifications table**

**Table:** 

Subject Area	Materials Science
More specific subject area	*In vitro models for ultrasound treatment; collagen scaffolds for cell culture*
Protocol name	*In vitro* cell culture models for ultrasound-based therapies*.*
Reagents/tools	*REAGENTS: NIH-3T3 fibroblast cells from ATCC (ATCC CRL-1658), Dulbecco's modified Eagle's medium (DMEM), fetal bovine serum(FBS), 0.05% trypsin-EDTA, 100X penicillin-streptomycin antibiotic solution, trypan blue and phosphate buffer saline (PBS) from Fisher Scientific, Fisherbrand sterile 12 well plates, Deionized water from Millipore System (MilliQ water), D-Glucose, L-Glutamine and 110 mg/L Sodium Pyruvate from Fisher Scientific, HEPES from Millipore Sigma, Penicillin and Streptomycin (P/S) from Invitrogen, Bovine tendon type I collagen from Organogenesis, Minimum essential medium with Earle's salts (MEM 10x), L-glutamine and sodium bicarbonate from Cambrex, alamarBlue from Fisher Scientific* *TOOLS: Ultrasound treatment using a 130-watt vibracell ultrasonic processor (VCX 130) with a 6 mm probe from Sonics & Materials INC, SpectraMax 2 plate reader from Molecular Devices.*
Experimental design	*2D and 3D collagen materials were utilized for cell culture for ultrasound treatment. For 2D cell culture, monolayer of cells were grown on collagen-coated substrates. For 3D cell culture, cells were suspended into collagen solution and cast to form hydrogels. Cells were allowed to continue growing for 7 days. Ultrasound treatment was conducted on all samples for varying durations of time before measuring cell viability using alamarBlue assay.*
Trial registration	*Not applicable*
Ethics	*Not applicable*
Value of the Protocol	Traditional cell cultures, unlike native tissue cannot protect cells during ultrasound treatment due to absence of extracellular matrix, leading to ∼90% cytotoxicity. In this protocol, collagen-based materials are utilized as ECM mimics to protect the cells and enable studies involving ultrasound treatment. We developed 2D and 3D cell culture models for ultrasound treatment using collagen coatings and collagen hydrogels respectively.

## Introduction

Ultrasound treatment has gained interest as a therapeutic strategy owing to its enhanced tissue penetration [1,2], versatile applications [3], [4], [5], [6], [7], easy accessibility [8,9], and minimally invasive procedures [10,11]. The frequency of ultrasound is easily modulated to localize desired biological effects such as thermal ablation of tissue, cavitation-induced cell permeability, and gas body activation [3]. Ultrasound activatable materials, such as alginate hydrogels, are utilized for developing drug-loadable scaffolds for triggerable and localized drug delivery [12], [13], [14]. More recently, sonodynamic therapy-based approaches have gained interest as potential antimicrobial and anti-cancer strategies where ultrasound is used in combination with sonosensitizers to trigger generation of ROS species [15], [16], [17]. Consequently, ultrasound treatment has been utilized for a variety of applications including – controlled and triggered drug release [12], [13], [14], sonodynamic therapy [15], [16], [17], targeted tissue ablation [18,19], and enhancing the penetration of drugs[29] [1,2].

However, the ability to study and explore new ultrasound-based therapeutic strategies is limited by the dearth of effective *in vitro* models that predict the effects of ultrasound-based treatments [20]. Cells grown *in vitro* often experience different biomechanical environments as compared to cells in native tissue [21]. In the native tissue, the extracellular matrix absorbs a significant amount of ultrasonic energy during treatment, thereby protecting the cells from lysis. In the absence of the ECM *in vitro*, cells are more prone to lysis [22]. This results in inconclusive results from *in vitro* studies and often animal models have to be utilized to assess the safety and efficacy of ultrasound. However, utilizing *in vivo* models for high throughput testing and screening is expensive and time consuming. For this reason, there is a need for effective *in vitro* models that mimic the mechanical environment of native tissue to enable translation of ultrasound-based therapeutic strategies

**Fig. 1:**
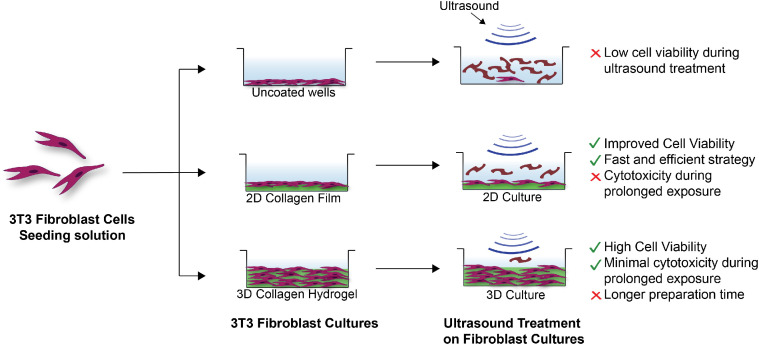
Schematic describing 3T3 Fibroblast cells cultured on (top to bottom) uncoated wells, 2D collagen films, and within 3D collagen hydrogels. Cells cultured on uncoated wells show uncontrolled and rapid cell lysis. Cells cultured on collagen films withstand some ultrasound exposure, while cells cultured within the 3D hydrogels withstand prolonged ultrasound exposure.

We utilized collagen-based materials to generate two-dimensional and three-dimensional cell culture models for ultrasound treatments. Collagen is a natural biopolymer and the primary component of the ECM in native tissues [23]. Therefore, it is inherently biocompatible and non-toxic [24]. Furthermore, there are numerous fabrication strategies available to generate collagen-based materials, such as films and hydrogels, with varying mechanical properties [25,26]. We hypothesized that the collagen matrix will mimic the ECM, absorbing the mechanical energy generated during ultrasound treatment thereby preserving the cells. We evaluated this using two different types of materials as a support for culturing 3T3 fibroblast cells – 2D collagen coatings and 3D collagen hydrogels [27,28]. Summarized below is the strategy for fabricating both types of scaffolds, culturing 3T3 cells and conducting ultrasound treatment, and evaluating cell viability. Our results indicated that both the 2D and 3D cell cultures are effective *in vitro* models with different advantages and limitations, as indicated in Fig. 1. The 2D collagen film is easy and quick to prepare and utilizes small amounts of materials. Therefore, this is ideal for preliminary studies and for testing a large number of conditions. However, it cannot withstand prolonged exposure to ultrasound. On the other hand, the 3D hydrogel effectively protects the cells from prolonged exposure to ultrasound and is more representative of native tissue. However, 3D cultures utilize significantly more material and take longer to fabricate (∼ 1 week). Overall, both strategies are potential *in vitro* models for testing ultrasound-based therapies and may be utilized for validating a variety of therapeutic applications.

**Fig. 2:**
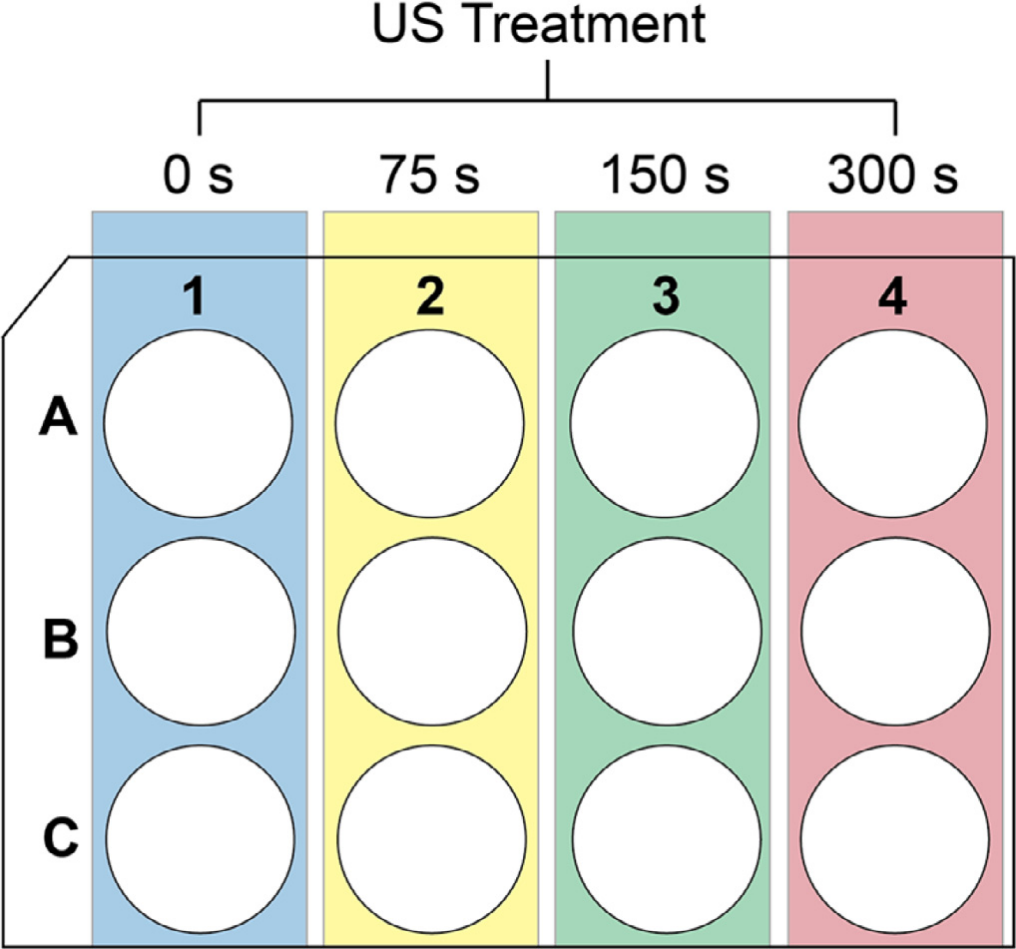
Schematic representation for conducting ultrasound treatment on a 12 well plate. Each treatment condition (yellow, green and red) were replicated three times. Blue column represents the untreated negative (growth) controls. A similar set up was utilized for uncoated or collagen-coated plates (2D cultures) or plates containing 3D collagen hydrogel cultures.

## Protocol 1 - 2D Cell Culture

*Step 1: Fabrication of 2D collagen coatings*

*Materials*

*Fisherbrand sterile 12 well plates (used for all cell cultures), Gibco rat tail collagen 1 solution (concentration 3mg/mL) and phosphate buffer saline (PBS) were purchased from Fisher Scientific. Deionized water produced by a Millipore System (MilliQ water) was utilized post sterilization by autoclaving. All procedures were performed under sterile conditions in a BSL 2 facility inside a biosafety cabinet.*

*Procedure*

1. Coatings were always prepared in a BSL 2 facility inside a biosafety cabinet to maintain sterile conditions at all times.
2. Coating solution was prepared by diluting the collagen 1 solution in milliQ water to a final concentration of 1 mg/mL. Sufficient solution was prepared to coat all the wells needed to perform the experiment.
3. Following this, 1 mL of the coating solution was placed in each well of a sterile 12 well plate. The plate was then incubated at 37 °C for 30 min to allow for the formation of collagen film. Appropriate number of wells were left uncoated for control groups.
4. The excess collagen solution was then removed, and the coated wells were washed three times using PBS.
5. Plates were then used immediately for cell culture.

*Step 2: Fibroblast culture on 2D collagen films*

*Materials*

*NIH-3T3 fibroblast cells were purchased from ATCC (ATCC CRL-1658). Dulbecco's modified Eagle's medium (DMEM), fetal bovine serum (FBS), 0.05% trypsin-EDTA, 100X penicillin-streptomycin antibiotic solution, trypan blue and phosphate buffer saline (PBS) were purchased from Fisher Scientific. DMEM was spiked with 10% v/v FBS and 1% v/v antibiotic solution to make the complete growth medium.*

*Procedure*

***Step 1: Thawing***

1. A frozen stock (∼1 mL) of NIH-3T3 fibroblast cells was thawed rapidly (<1 minute) in a 37°C water bath.
2. Thawed cells were washed by diluting with 9 mL pre-warmed complete growth medium and then centrifuged at 3000rpm for 5 min to prepare a cell pellet.
3. The growth medium was then removed without disturbing the pellet and the cells were redispersed in 9 mL fresh growth medium to prepare the cell solution.
4. This cell solution was then transferred completely to a T-75 culture flask for further culturing.

***Step 2: Cell culture and cell counting.***

1. Once the cells were 80-90% confluent, the growth medium was removed and washed with PBS.
2. The cells were then trypsinized with ∼3mL of 0.05 % Trypsin-EDTA and incubated at 37°C for 5-7 mins to allow the cells to detach.
3. Once the cells were detached, 7mL of complete medium was added to neutralize the trypsin.
4. The cell solution was then collected in a 15mL Falcon tube and centrifuged at 3000rpm for 5 minutes to collect the cell pellet.
5. The growth medium was removed without disturbing the pellet and resuspended with fresh complete growth medium accordingly.
6. 10uL of cell solution was taken in an Eppendorf tube and mixed with 10uL of trypan blue. 10uL of this mixture was then added to one side of the disposable cell counting chamber slide and inserted into the Invitrogen Countess II Automated Cell Counter machine to count the number of cells in the cell solution.

***Step 3: Cell Plating.***

The cell solution was diluted to 100,000 cells/well and plated on uncoated or collagen-coated wells in a 12-well plate and allowed to grow overnight at 37°C in a humidified atmosphere of 5% CO2 and were treated with ultrasound the following day.

***Step 4: Cell Splitting.***

The remaining cell solution was split in a ratio of 1:10 into a new culture flask and sub-cultured every 2 days for further experiments.

## Protocol 2 - 3D Cell Culture

*Materials*

*Fisherbrand sterile 12 well plates (used for all cell cultures) and phosphate buffer saline (PBS) were purchased from Fisher Scientific. Deionized water produced by a Millipore System (MilliQ water) was utilized post sterilization by autoclaving. Fetal Bovine Serum (FBS) was purchased from Peak Serum. Dulbecco's Modified Eagle Medium 1x (DMEM) with 4.5 g/L D-Glucose, L-Glutamine and 110 mg/L Sodium Pyruvate was purchased from Fisher Scientific. HEPES solution was purchased from Millipore Sigma. Penicillin and Streptomycin (P/S) were purchased from Invitrogen. Bovine tendon type I collagen was purchased from Organogenesis. Minimum essential medium with Earle's salts (MEM 10x), L-glutamine and sodium bicarbonate was purchased from Cambrex. All reagents were utilized as obtained from the vendor without additional purification, unless otherwise specified. All procedures were performed under sterile conditions in a BSL 2 facility inside a biosafety cabinet.*

*Procedure*

The protocol described in ref 28 was utilized for fabricating the 3D cell cultures.

***Step 1: Preparation of components 24 hr prior***

1. The conditioned media was prepared by using Gibco's DMEM 1x and mixing it with 10% FBS, 1% P/S and 1% HEPES under sterile conditions.
2. The following were placed in the refrigerator at 4 °C 24 hr before fabrication of gels - FBS, glutamine, DMEM, sodium bicarbonate, pipette tips (p1000, p200), 15 mL Falcon tubes, and 12 well culture plates.
3. Sodium bicarbonate (1M) was sterilized using a PTFE syringe filter with pore size 0.22um. Forceps were sterilized by autoclaving prior to use.
4. Cells were thawed and cultured in a T-75 flask through the procedure described above and grown to 80-90% confluency before the next step.

***Step 2: Preparation of the cell seeding solution.***

1. Once the cells reached 80-90% confluency, the growth medium was carefully extracted from the flask and replaced with 5 mL of PBS that was warmed in a water bath at 37 °C.
2. Cells were then washed by gentle swirling and the PBS was carefully removed and discarded from the flask.
3. Following this, 2 mL of 0.05% trypsin-EDTA was added to the flask and incubated at 37°C for 8-10 minutes to detach the cells.
4. Once the cells were detached, ∼ 6 mL of conditioned DMEM medium was added to neutralize the trypsin and dilute the solution.
5. The cell solution was then placed in a 15 mL Falcon tube and centrifuged at 1,000 rpm for 10 min at 4 °C.
6. Excess medium was carefully extracted without disturbing the cell pellet and the cells were resuspended in 15 mL of fresh growth medium.
7. 10 μL of cell solution from above was added to a sterile 1.5 mL Eppendorf tube along with 40 μL of fresh growth DMEM medium and 50 μL of Trypan Blue. Cells were resuspended and 10 μL was extracted and placed on a hemocytometer for counting.
8. Finally, 3.22 mL of cell seeding solution was prepared at the concentration of 300,000 cells/ mL.

***Step 3: Casting 3D collagen gels***

1. The cell seeding solution, growth medium, two 50 mL Falcon tubes, collagen 1 solution, sterile NaHCO_3_ solution, 12 well plates, and transfer pipets were all placed in an ice bath to maintain low temperature. Care was taken to keep all components cold during the experiment.
2. One of the two Falcon tubes were used to prepare the cell-collagen solution (Tube 1) while the other was used to prepare the cell-free control collagen solution (Tube 2). Each tube was prepared with the components described in Table 1 and mixed well by pipetting back and forth several times. The final solution should be a straw yellow color or darker. If lighter, more NaHCO_3_ solution was added in 80 μL increments until the desired color was formed.

**Table 1:** Table summarizes volume of each component added to Tube 1 and Tube 2 to prepare 18 and 4 gels respectively. Components must be adjusted to prepare more gels while keeping the ratio same.

Components	Amount in Tube 1	Amount in Tube 2
10 x MEM	3.5 mL	875 μL
L - Glutamine	316 μL	79 μL
FBS	3.92 mL	980 μL
NaHCO3 solution	1089 μL	273 μL
Collagen	29.17 mL	7.3 mL
3. **To Tube 1**, 3.22 mL of the cell seeding solution was added after thorough mixing. The contents of Tube 1 were then mixed well by pipetting back and forth several times. Following this 2 mL of solution was placed in each well of a 12 well plate. This solution formed about 18 gels. Care was taken to minimize formation of bubbles during mixing.
4. **To Tube 2,** 804 μL of DMEM was added to prepare the cell-free control gels. The solution was mixed well as described above and 2 mL of the solution was placed in each well of a 12 well plate. This solution formed about 4 gels.
5. Gels were incubated at 37 °C and 7.5% CO_2_ for 1 hr, to firm up. A pink coloration was observed when the gels were firm.
6. The cells were fed with 2 mL of warm growth medium every other day for a total of 7 days before proceeding with ultrasound treatment.

## Protocol 3 - Ultrasound Treatment

*Equipment*

*Ultrasound treatment was done using a 130-watt vibracell ultrasonic processor (VCX 130) with a 6 mm probe purchased from Sonics & Materials INC and was utilized at 35% amplitude for the time durations specified.*

*Procedure*

1. Each well was topped off with enough media to ensure a final volume of 2 mL.
2. The ultrasound probe was dipped into the center of the well to ∼ 1-2 mm depth. This is to ensure proper ultrasound exposure throughout the treatment duration.
3. Ultrasound treatments were done for 75, 150 and 300 seconds on cells cultured on both the 2D and 3D cultures. Each ultrasound treatment condition was repeated on at least 3 different wells. At least 3 wells in each culture were left untreated to serve as negative (growth) controls. See Fig. 1 for experimental set up.
4. Step 3 was repeated on uncoated 12 well plates were also treated with 75, 150 and 300 seconds of ultrasound treatment to serve as positive controls.

## Protocol 4 – Determination of Cell Viability Post Ultrasound Treatment

*Materials and Equipment*

*The Invitrogen alamarBlue dye was purchased from Fisher Scientific. Fluorescence signal was measured on a SpectraMax M2 plate reader from Molecular Devices.*

*Procedure*

1. The protocol provided by the manufacturer for planktonic cells was utilized. This was done because ultrasound treatment may displace adherent cells but not necessarily result in cell death. Therefore, this strategy was able to measure all viable cells even if they are not adhered to the bottom of the well post treatment.
2. After the US treatment, each cell culture well of the 12 well plates was spiked with 200 μL of alamarBlue solution. The solution in each well was mixed by gently pipetting back and forth a few times and all the plates were incubated at 37 °C for 3 hours.
3. Simultaneously, 6 wells on a black 96 well plate were filled with 100 μL of the same DMEM media used for cell culture, spiked with 10 μL of alamarBlue solution, mixed well, and incubated at 37 °C for 3 hours along with the cell culture plates. This will act as the reference value for the baseline signal from the media, when calculating the cell viability.
4. After 3 hours, 330 μL of supernatant was removed from each cell culture well (from step 2) and placed into three separate wells (110 μL each) of the black 96 well plate with the reference prepared in step 3. This ensures that each cell culture well is being measured at least 3 times.
5. As each treatment condition (described in the section - procedure for ultrasound treatment) was replicated 3 times and each cell culture well was sampled 3 times (described in step 4), the final measurements include 9 measurements per treatment condition. The final measurements must also include 9 measurements from both the positive and negative controls described previously.
6. Finally, the fluorescence signal from each well of the black 96 well plate was measured at excitation wavelength of 560 nm and emission of 590 nm.
7. % Cell viability was calculated for each treatment condition using the formula –
  $$\%\mathrm{Cell}\;\mathrm{Viab}\mathrm{ility}=\frac{\mathrm{Fluo}\mathrm{resc}\mathrm{ence}\;\mathrm{sign}\mathrm{al}\;\mathrm{from}\;\mathrm{trea}\mathrm{ted}\;\mathrm{well}-\mathrm{Fluo}\mathrm{resc}\mathrm{ence}\;\mathrm{sign}\mathrm{al}\;\mathrm{from}\;\mathrm{refe}\mathrm{rence}}{\mathrm{Fluo}\mathrm{resc}\mathrm{ence}\;\mathrm{sign}\mathrm{al}\;\mathrm{from}\;\mathrm{untr}\mathrm{eated}\;\left(\mathrm{grow}\mathrm{th}\right)\mathrm{cont}\mathrm{rol}-\mathrm{Fluo}\mathrm{resc}\mathrm{ence}\;\mathrm{sign}\mathrm{al}\;\mathrm{from}\;\mathrm{refe}\mathrm{rence}}\times 100$$
8 Average %Cell viability and standard deviation were obtained from the replicates described in step 5 and reported in Method Validation. This protocol was repeated two additional times to ensure reproducibility.

## Protocol Validation

**Fig. 3:**
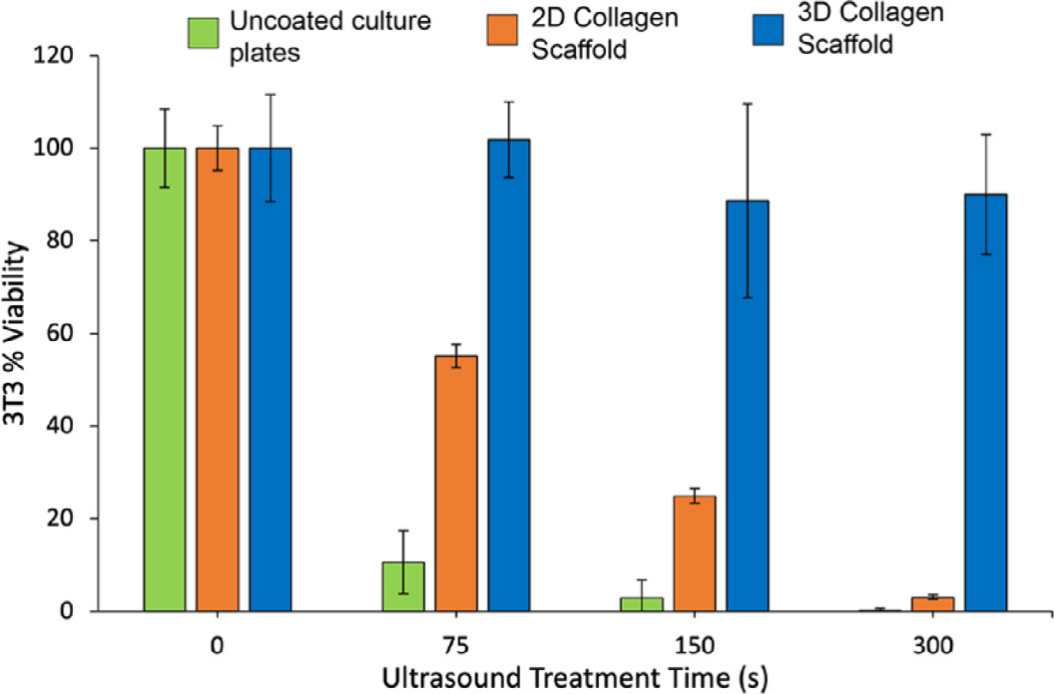
%Cell viability post ultrasound treatment for cells grown on uncoated plates (green), plates containing 2D collagen scaffold (orange), and plates containing 3D collagen scaffold (blue). 2D scaffold results in ∼40% increase in cell viability while 3D scaffold results in ∼90% increase.

Fig. 3 shows the %cell viability for different durations of ultrasound treatment for 2D cell cultures on uncoated and collagen coated substrates as well as a 3D cell culture in collagen hydrogels. As seen in Fig. 3, cells grown on uncoated plates (green bars) show minimal resilience against ultrasound, with only ∼10% of the cells surviving after 75 s of ultrasound treatment. By comparison, the 2D collagen scaffold (orange bars) increases the cell viability dramatically, with over 50% cells surviving 75 s of ultrasound treatment. However, prolonged treatment results in increased cell death. In the case of the 3D collagen hydrogels, > 80% of the cells survive even after 300 s of ultrasound treatment (as seen by the blue bars).

## Conclusion

We therefore concluded that collagen-based materials significantly increase the resilience of cells against ultrasound treatment by mimicking the extracellular matrix in tissue and absorbing some of the mechanical energy generated by the ultrasound. The 2D collagen scaffold offers a rapid and efficient way to test ultrasound-based treatments, as the experiment can be conducted within 24 hr and requires only a small amount of material. This strategy is therefore ideal for preliminary experiments or experiments with short duration of ultrasound exposure (∼1 min). On the contrary, 3D cultures take ∼1 week and significantly higher amounts of collagen to fabricate. However, the 3D cultures provide significantly more protection from prolonged exposure to ultrasound treatment and are therefore more representative of native tissue. Taken together, these collagen-based 2D and 3D cultures have the potential to serve as *in vitro* model for testing ultrasound-based treatment strategies for a variety of applications such as controlled drug delivery, anti-cancer, and antimicrobial strategies.

## Declaration of Competing Interest

The authors declare that they have no known competing financial interests or personal relationships that could have appeared to influence the work reported in this paper.

## Data Availability

Data will be made available on request.
